# Supplementary material for: Intron gain by tandem genomic duplication: a novel case in a potato gene encoding RNA-dependent RNA polymerase
Source: PeerJ. 2016 Jul 26;4:e2272. doi: 10.7717/peerj.2272 (PMC4974935; doi:10.7717/peerj.2272)
Supplement: Supplemental Information 2 — Table S1: The RNA-Seq reads mapped crossing the exon-exon boundary of the target intron; Table S2: The WGS reads mapped crossing the boundaries of the two duplicates. [file peerj-04-2272-s002.docx]

Supplemental Information 2 of

**Intron gain by tandem genomic duplication: a novel case in a potato gene encoding RNA-dependent RNA polymerase**

Ming-Yue Ma, Xin-Ran Lan and Deng-Ke Niu

Figure S1. Positions of the annotated *RdRp* isoforms on potato chromosome 12. The boxes represent exons and the horizontal lines represent introns. The coding sequences are marked in blue and the untranslated regions are marked in gray. *PGSC0003DMG402000361.S* and *PGSC0003DMG402000361.L* have been confirmed by RNA-Seq reads. *PGSC0003DMG401000361* was retrieved from the annotation files of the domesticated potato genome PGSC_DM_v3. Because *PGSC0003DMG401000361* is not relevant to the intron gain event, we did not examine its RNA-Seq data and thus cannot guarantee its reliability.





Figure S2. RNA-Seq reads mapped crossing the exon-exon boundary of the target intron. The exact nucleotide sequences in this figure are visible when it is viewed at the 400% zoom setting.

**Target sequences GTGACAACTAGCTTTCAAAGAGATCATTACGATCCTAGGCCATCTACATTCAGAGACAGGGCCAGCACACGAGGAATAAGTGAACAGTTGCTGGCACTCAgtaagcttgaattagtttaggcttaatgaagaacttgttcaatttttttattggttgcgatctcttcttcctttttttgcatatttacaactctacatgtaaactatgttgctcggactctcaaaaactgttgaacccgtgttggattctccaaaatgcactacttttggagtattcgatacacacttttgaagagtccgaacaacacaacatgtaatgtactcagacctttcaagaattctagtttaccaatgatggtcttccaatgcctgcagACATAGTAGGTGATGCATCTGATTCTCCTACATCAGCTCCACGAATACCATCACCTCCAATGAGTCCAGTGACAACTAGCTTTCAAAGAGATCATTACGA**

**ERR305631.sra.5633486 ----------------------------------------CATCTACATTCAGAGACAGGGCCAGCATACGAGGAATAAGTGAACAGTTGCTGGCACTCA-----------------------------------------------------------------------------------------------------------------------------------------------------------------------------------------------------------------------------------------------------------------------------------ACACAGTAGGTGATGCATCTGATTCTCCTA----------------------------------------------------------------------**

**ERR305631.sra.14764201 -----------------------------------------ATCTACATTCAGAGACAGGGCCAGCACACGAGGAATAAGTGAACAGTTGCTGGCACTCA-----------------------------------------------------------------------------------------------------------------------------------------------------------------------------------------------------------------------------------------------------------------------------------ACATAGTAGGTGATGCATCTGATTCTCCTAC---------------------------------------------------------------------**

**ERR305631.sra.1954676 -----------------------------------------------ATTCAGAGACAGGGCCAGCACACGAGGAATAAGTGAACAGTTGCTGGCACTCA-----------------------------------------------------------------------------------------------------------------------------------------------------------------------------------------------------------------------------------------------------------------------------------ACATAGTAGGTGATGCATCTGATTCTCCTACATCAGC---------------------------------------------------------------**

**ERR305631.sra.25528009 -----------------------------------------------ATTCAGAGACAGGGCCAGCACACGAGGAATAAGTGAACAGTTGCTGGCACTCA-----------------------------------------------------------------------------------------------------------------------------------------------------------------------------------------------------------------------------------------------------------------------------------ACATAGTAGGTGATGCATCTGATTCTCCTACATCAGC---------------------------------------------------------------**

**ERR305631.sra.11034325 -----------------------------------------------------AGACAGGGCCAGCATACGAGGAATAAGTGAACAGTTGCTGGCACTCA-----------------------------------------------------------------------------------------------------------------------------------------------------------------------------------------------------------------------------------------------------------------------------------ACACAGTAGGTGATGCATCTGATTCTCCTACATCAGCTCCACG---------------------------------------------------------**

**ERR305631.sra.21174123 ------------------------------------------------------GACAGGGCCAGCATACGAGGAATAAGTGAACAGTTGCTGGCACTCA-----------------------------------------------------------------------------------------------------------------------------------------------------------------------------------------------------------------------------------------------------------------------------------ACACAGTAGGTGATGCATCTGATTCTCCTACATCAGCTCCACGA--------------------------------------------------------**

**ERR305631.sra.14962990 ----------------------------------------------------------GGGCCAGCATACGAGGAATAAGTGAACAGTTGCTGGCACTCA-----------------------------------------------------------------------------------------------------------------------------------------------------------------------------------------------------------------------------------------------------------------------------------ACACAGTAGGTGATGCATCTGATTCTCCTACATCAGCTCCACGAATAC----------------------------------------------------**

**ERR305631.sra.1208780 ------------------------------------------------------------GCCAGCATACGAGGAATAAGTGAACAGTTGCTGGCACTCA-----------------------------------------------------------------------------------------------------------------------------------------------------------------------------------------------------------------------------------------------------------------------------------ACACAGTAGGTGATGCATCTGATTCTCCTACATCAGCTCCACGAATACCA--------------------------------------------------**

**ERR305631.sra.19438135 -------------------------------------------------------------------------------GTGAACAGTTGCTGGCACTCA-----------------------------------------------------------------------------------------------------------------------------------------------------------------------------------------------------------------------------------------------------------------------------------ACATAGTAGGTGATGCATCTGATTCTCCTACATCAGCTCCACGAATACCATCACCTCCAATGAGTCCAG-------------------------------**

**ERR305631.sra.21551313 -------------------------------------------------------------------------------------AGTTGCTGGCACTCA-----------------------------------------------------------------------------------------------------------------------------------------------------------------------------------------------------------------------------------------------------------------------------------ACATAGTAGGTGATGCATCTGATTCTCCTACATCAGCTCCACGAATACCATCACCTCCAATGAGTCCAGTGACAA-------------------------**

**ERR305632.sra.5125036 -----------------AAGAGATCATTACGATCCTAGGCCATCTACATTCAGAGACAGGGCCAGCACACGAGGAATAAGTGAACAGTTACTGGCACTCA-----------------------------------------------------------------------------------------------------------------------------------------------------------------------------------------------------------------------------------------------------------------------------------ACATAGT---------------------------------------------------------------------------------------------**

**ERR305632.sra.17798760 -----------------------------CGATCCTAGGCCATCTACATTCAGAGACAGGGCCAGCACACGAGGAATAAGTGAACAGTTGCTGGCACTCA-----------------------------------------------------------------------------------------------------------------------------------------------------------------------------------------------------------------------------------------------------------------------------------ACATAGTAGGTGATGCATC---------------------------------------------------------------------------------**

**ERR305632.sra.19889662 ------------------------------------AGGCCATCTACATTCAGAGACAGGGCCAGCACACGAGGAATAAGTGAACAGTTGCTGGCACTCA-----------------------------------------------------------------------------------------------------------------------------------------------------------------------------------------------------------------------------------------------------------------------------------ACATAGTAGGTGATGCATCTGATTCT--------------------------------------------------------------------------**

**ERR305632.sra.23851887 ------------------------------------AGGCCATCTACATTCAGAGACAGGGCCAGCACACGAGGAATAAGTGAACAGTTGCTCGCACTCA-----------------------------------------------------------------------------------------------------------------------------------------------------------------------------------------------------------------------------------------------------------------------------------ACATAGTAGGTGATGCATCTGATTCT--------------------------------------------------------------------------**

**ERR305632.sra.22237812 -------------------------------------GGCCATCTACATTCAGAGACAGGGCCAGCACACGAGGAATAAGTGAACAGTTGCTGGCACTCA-----------------------------------------------------------------------------------------------------------------------------------------------------------------------------------------------------------------------------------------------------------------------------------ACATAGTAGGTGATGCATCTGATTCTC-------------------------------------------------------------------------**

**ERR305632.sra.17496368 --------------------------------------GCCATCTACATTCAGAGACAGGGCCAGCACACGAGGAATAAGTGAACAGTTGCTGGCACTCA-----------------------------------------------------------------------------------------------------------------------------------------------------------------------------------------------------------------------------------------------------------------------------------ACATAGTAGGTGATGCATCTGATTCTCC------------------------------------------------------------------------**

**ERR305632.sra.20019441 ------------------------------------------------------------------------------------------CTGGCACTCA-----------------------------------------------------------------------------------------------------------------------------------------------------------------------------------------------------------------------------------------------------------------------------------ACATAGTAGGTGATGCATCTGATTCTCCTACATCAGCTCCACGAATACCATCACCTCCAATGAGTCCAGTGACAACTAGC--------------------**

**SRR1207283.sra.9962581 ---------------------------TACGATCCTAGGCCATCTACATTCAGAGACAGGGCCAGCACACGAGGAATAAGAGAACAGTTGCTGGCACTCA-----------------------------------------------------------------------------------------------------------------------------------------------------------------------------------------------------------------------------------------------------------------------------------ACATAGTAGGTGATGCA-----------------------------------------------------------------------------------**

**SRR1207283.sra.23466160 -----------------------------CGATCCTAGGCCATCTACATTCAGAGACAGGGCCAGCACACGAGGAATAAGTGAACAGTTGCTGGCACTCA-----------------------------------------------------------------------------------------------------------------------------------------------------------------------------------------------------------------------------------------------------------------------------------ACATAGTAGGTGATGCATC---------------------------------------------------------------------------------**

**SRR1207283.sra.21759433 ----------------------------------CTAGGCCATCTACATTCAGAGACAGGGCCAGCACACGAGGAATAAGTGAACAGTTGCTGGCACTCA-----------------------------------------------------------------------------------------------------------------------------------------------------------------------------------------------------------------------------------------------------------------------------------ACATAGTAGGTGATGCATCTGATT----------------------------------------------------------------------------**

**SRR1207283.sra.22329356 ----------------------------------CTAGGCCATCTACATTCAGAGACAGGGCCAGCACACGAGGAATAAGTGAACAGTTGCTGGCACTCA-----------------------------------------------------------------------------------------------------------------------------------------------------------------------------------------------------------------------------------------------------------------------------------ACATAGTAGGTGATGCATCTGATT----------------------------------------------------------------------------**

**SRR1207283.sra.5226164 -------------------------------------GGCCATCTACATTCAGAGACAGGGCCAGCATACGAGGAATAAGTGAACAGTTGCTGGCACTCA-----------------------------------------------------------------------------------------------------------------------------------------------------------------------------------------------------------------------------------------------------------------------------------ACACAGTAGGTGATGCATCTGATTCTC-------------------------------------------------------------------------**

**SRR1207283.sra.22364697 --------------------------------------GCCATCTACATTCAGAGACAGGGCCAGCACACGAGGAATAAGTGAACAGTTGCTGGCACTCA-----------------------------------------------------------------------------------------------------------------------------------------------------------------------------------------------------------------------------------------------------------------------------------ACATAGTAGGTGATGCATCTGATTCTCC------------------------------------------------------------------------**

**SRR1207283.sra.24433899 --------------------------------------GCCATCTACATTCAGAGACAGGGCCAGCACACGAGGAATAAGTGAACAGTTGCTGGCACTCA-----------------------------------------------------------------------------------------------------------------------------------------------------------------------------------------------------------------------------------------------------------------------------------ACATAGTAGGTGATGCATCTGATTCTCC------------------------------------------------------------------------**

**SRR1207283.sra.22477210 --------------------------------------------------CAGAGACAGGGCCAGCATACGAGGAATAAGTGAACAGTTGCTGGCACTCA-----------------------------------------------------------------------------------------------------------------------------------------------------------------------------------------------------------------------------------------------------------------------------------ACACAGTAGGTGATGCATCTGATTCTCCTACATCAGCTCC------------------------------------------------------------**

**SRR1207283.sra.3800679 ---------------------------------------------------AGAGACAGGGCCAGCACACGAGGAATAAGTGAACAGTTGCTGGCACTCA-----------------------------------------------------------------------------------------------------------------------------------------------------------------------------------------------------------------------------------------------------------------------------------ACATAGTAGGTGATGCATCTGATTCTCCTACATCAGCTCCA-----------------------------------------------------------**

**SRR1207283.sra.5373704 ----------------------------------------------------GAGACAGGGCCAGCACACGAGGAATAAGTGAACAGTTGCTGGCACTCA-----------------------------------------------------------------------------------------------------------------------------------------------------------------------------------------------------------------------------------------------------------------------------------ACATAGTAGGTGATGCATCTGATTCTCCTACATCAGCTCCAC----------------------------------------------------------**

**SRR1207283.sra.4383324 -----------------------------------------------------AGACAGGGCCAGCACACGAGGAATAAGTGAACAGTTGCTGGCACTCA-----------------------------------------------------------------------------------------------------------------------------------------------------------------------------------------------------------------------------------------------------------------------------------ACATAGTAGGTGATGCATCTGATTCTCCTACATCAGCTCCACG---------------------------------------------------------**

**SRR1207283.sra.18821448 -----------------------------------------------------AGACAGGGCCAGCACACGAGGAATAAGTGAACAGTTGCTGGCACTCA-----------------------------------------------------------------------------------------------------------------------------------------------------------------------------------------------------------------------------------------------------------------------------------ACATAGTAGGTGATGCATCTGATTCTCCTACATCAGCTCCACG---------------------------------------------------------**

**SRR1207283.sra.19790545 --------------------------------------------------------------------------------------GTTGCTGGCACTCA-----------------------------------------------------------------------------------------------------------------------------------------------------------------------------------------------------------------------------------------------------------------------------------ACATAGTAGGTGATGCATCTGATTCTCCTACATCAGCTCCACGAATACCATCACCTCCAATGAGTCCAGTGACAAC------------------------**

**SRR1207283.sra.15936888 ------------------------------------------------------------------------------------------CTGGCACTCA-----------------------------------------------------------------------------------------------------------------------------------------------------------------------------------------------------------------------------------------------------------------------------------ACATAGTAGGTGATGCATCTGATTCTCCTACATCAGCTCCACGAATACCATCACCTCCAATGAGTCCAGTGACAACTAGC--------------------**

**SRR1207284.sra.6983677 ---------------CAAAGAGATCATTACGATCCTAGGCCATCTACATTCAGAGACAGGGCCAGCACACGAGGAATAAGTGAACAGTTGCTGGCACTCA-----------------------------------------------------------------------------------------------------------------------------------------------------------------------------------------------------------------------------------------------------------------------------------ACATA-----------------------------------------------------------------------------------------------**

**SRR1207284.sra.13221004 ---------------CAAAGAGATCATTACGATCCTAGGCCATCTACATTCAGAGACAGGGCCAGCACACGAGGAATAAGTGAACAGTTGCTGGCACTCA-----------------------------------------------------------------------------------------------------------------------------------------------------------------------------------------------------------------------------------------------------------------------------------ACATA-----------------------------------------------------------------------------------------------**

**SRR1207284.sra.737394 -------------------GAGATCATTACGATCCTAGGCCATCTACATTCAGAGACAGGGCCAGCACACGAGGAATAAGTGAACAGTTGCTGGCACTCA-----------------------------------------------------------------------------------------------------------------------------------------------------------------------------------------------------------------------------------------------------------------------------------ACATAGTAG-------------------------------------------------------------------------------------------**

**SRR1207284.sra.8745191 ------------------------CATTACGATCCTAGGCCATCTACATTCAGAGACAGGGCCAGCACACGAGGAATAAGTGATCAGTTGCTGGCACTCA-----------------------------------------------------------------------------------------------------------------------------------------------------------------------------------------------------------------------------------------------------------------------------------ACATAGTAGGTGAT--------------------------------------------------------------------------------------**

**SRR1207284.sra.11182140 -------------------------ATTACGATCCTAGGCCATCTACATTCAGAGACAGGGCCAGCACACGAGGAATAAGTGAACAGTTGCTGGCACTCA-----------------------------------------------------------------------------------------------------------------------------------------------------------------------------------------------------------------------------------------------------------------------------------ACATAGTAGGTGATG-------------------------------------------------------------------------------------**

**SRR1207284.sra.15679219 --------------------------TTACGATCCTAGGCCATCTACATTCAGAGACAGGGCCAGCACACGAGGAATAAGTGAACAGTTGCTGGCACTCA-----------------------------------------------------------------------------------------------------------------------------------------------------------------------------------------------------------------------------------------------------------------------------------ACATAGTAGGTGATGC------------------------------------------------------------------------------------**

**SRR1207284.sra.12578766 ---------------------------TACGATCCTAGGCCATCTACATTCAGAGACAGGGCCAGCACACGAGGAATAAGTGAACAGTTGCTGGCACTCA-----------------------------------------------------------------------------------------------------------------------------------------------------------------------------------------------------------------------------------------------------------------------------------ACATAGTAGGTGATGCA-----------------------------------------------------------------------------------**

**SRR1207284.sra.3184424 ------------------------------------AGGCCATCTACATTCAGAGACAGGGCCAGCACACGAGGAATAAGTGAACAGTTGCTGGCACTCA-----------------------------------------------------------------------------------------------------------------------------------------------------------------------------------------------------------------------------------------------------------------------------------ACATAGTAGGTGATGCATCTGATTCT--------------------------------------------------------------------------**

**SRR1207284.sra.21163685 ------------------------------------AGGCCATCTACATTCAGAGACAGGGCCAGCACACGAGGAATAAGTGAATAGTTGCTGGCACTCA-----------------------------------------------------------------------------------------------------------------------------------------------------------------------------------------------------------------------------------------------------------------------------------ACATAGTAGGTGATGCATCTGATTCT--------------------------------------------------------------------------**

**SRR1207284.sra.12838668 ----------------------------------------CATCTACATTCAGAGACAGGGCCAGCACACGAGGAATAAGTGAACAGTTGCTGGCACTCA-----------------------------------------------------------------------------------------------------------------------------------------------------------------------------------------------------------------------------------------------------------------------------------ACATAGTAGGTGATGCATCTGATTCTCCTA----------------------------------------------------------------------**

**SRR1207284.sra.15602843 ------------------------------------------------TTCAGAGACAGGGCCAGCACACGAGGAATAAGTGAACAGTTGCTGGCACTCA-----------------------------------------------------------------------------------------------------------------------------------------------------------------------------------------------------------------------------------------------------------------------------------ACATAGTAGGTGATGCATCTGATTCTCCTACATCAGCT--------------------------------------------------------------**

**SRR1207284.sra.308417 ----------------------------------------------------GAGACAGGGCCAGCACACGAGGAATAAGTGAACAGTTGCTGGCACTCA-----------------------------------------------------------------------------------------------------------------------------------------------------------------------------------------------------------------------------------------------------------------------------------ACATAGTAGGTGATGCATCTGATTCTCCTACATCAGCTCCAC----------------------------------------------------------**

**SRR1207284.sra.20128638 ----------------------------------------------------------------GCACACGAGGAATAAGTGAACAGTTGCTGGCACTCA-----------------------------------------------------------------------------------------------------------------------------------------------------------------------------------------------------------------------------------------------------------------------------------ACATAGTAGGTGATGCATCTGATTCTCCTACATCAGCTCCACGAATACCATCAC----------------------------------------------**

**SRR1207284.sra.2009732 ------------------------------------------------------------------------------AGTGAACAGTTGCTGGCACTCA-----------------------------------------------------------------------------------------------------------------------------------------------------------------------------------------------------------------------------------------------------------------------------------ACACAGTAGGTGATGCATCTGATTCTCCTACATCAGCTCCACGAATACCATCACATCCAATGAGTCCA--------------------------------**

**SRR1207284.sra.7527762 ------------------------------------------------------------------------------AGTGAACAGTTGCTGGCACTCA-----------------------------------------------------------------------------------------------------------------------------------------------------------------------------------------------------------------------------------------------------------------------------------ACACAGTAGGTGATGCATCTGATTCTCCTACATCAGCTCCACGAATACCATCACATCCAATGAGTCCA--------------------------------**

**SRR1207285.sra.19310059 ---------------------GATCATTACGATCCTAGGCCATCTACATTCAGAGACAGGGCCAGCACACGAGGAATAAGTGAACAGTTGCTGGCACTCA-----------------------------------------------------------------------------------------------------------------------------------------------------------------------------------------------------------------------------------------------------------------------------------ACATAGTAGGT-----------------------------------------------------------------------------------------**

**SRR1207285.sra.5814346 --------------------------TTACGATCCTAGGCCATCTACATTCAGAGACAGGGCCAGCACACGAGGAATAAGTGAAAAGTTGCTGGCACTCA-----------------------------------------------------------------------------------------------------------------------------------------------------------------------------------------------------------------------------------------------------------------------------------ACATAGTAGGTGATGC------------------------------------------------------------------------------------**

**SRR1207285.sra.20492630 ----------------------------------------------CATTCAGAGACAGGGCCAGCACACGAGGAATAAGTGAACAGTTGCTGGCACTCA-----------------------------------------------------------------------------------------------------------------------------------------------------------------------------------------------------------------------------------------------------------------------------------ACATAGTAGGTGATGCATCTGATTCTCCTACATCAG----------------------------------------------------------------**

**SRR1207285.sra.15254802 -----------------------------------------------ATTCAGAGACAGGGCCAGCACACGAGGAATAAGTGAACAGTTGCTGGCACTCA-----------------------------------------------------------------------------------------------------------------------------------------------------------------------------------------------------------------------------------------------------------------------------------ACATAGTAGGTGATGCATCTGATTCTCCTACATCAGC---------------------------------------------------------------**

**SRR1207285.sra.9590300 -----------------------------------------------ATTCAGAGACAGGGCCAGCACACGAGGAATAAGTGAACAGTTGCTGGCACTCA-----------------------------------------------------------------------------------------------------------------------------------------------------------------------------------------------------------------------------------------------------------------------------------ACATAGTAGGTGATGCATCTGATTCTCCTACATCAGC---------------------------------------------------------------**

**SRR1207285.sra.20691870 -----------------------------------------------ATTCAGAGACAGGGCCAGCACACGAGGAATAAGTGAACAGTTGCTGGCACTCA-----------------------------------------------------------------------------------------------------------------------------------------------------------------------------------------------------------------------------------------------------------------------------------ACATAGTAGGTGATGCATCTGATTCTCCTACATCAGC---------------------------------------------------------------**

**SRR1207285.sra.18633958 ----------------------------------------------------GAGACAGGGCCAGCATACGAGGAATAAGTGAACAGTTGCTGGCACTCA-----------------------------------------------------------------------------------------------------------------------------------------------------------------------------------------------------------------------------------------------------------------------------------ACACAGTAGGTGATGCATCTGATTCTCCTACATCAGCTCCAC----------------------------------------------------------**

**SRR1207285.sra.22438572 ------------------------------------------------------GACAGGGCCAGCACACGAGGAATAAGTGAACAGTTGCTGGCACTCA-----------------------------------------------------------------------------------------------------------------------------------------------------------------------------------------------------------------------------------------------------------------------------------ACATAGTAGGTGATGCATCTGATTCTCCTACATCAGCTCCACGG--------------------------------------------------------**

**SRR1207285.sra.59217 ----------------------------------------------------------GGGCCAGCACACGAGGAATAAGTGAACAGTTGCTGGCACTCA-----------------------------------------------------------------------------------------------------------------------------------------------------------------------------------------------------------------------------------------------------------------------------------ACATAGTAGGTGATGCATCTGATTCTCCTACATCAGCTCCACGAATAC----------------------------------------------------**

**SRR1207285.sra.16151887 ----------------------------------------------------------------GCATACGAGGAATAAGTGAACAGTTGCTGGCACTCA-----------------------------------------------------------------------------------------------------------------------------------------------------------------------------------------------------------------------------------------------------------------------------------ACACAGTAGGTGATGCATCTGATTCTCCTACATCAGCTCCACGAATACCATCAC----------------------------------------------**

**SRR1207285.sra.21592470 ----------------------------------------------------------------GCATACGAGGAATAAGTGAACAGTTGCTGGCACTCA-----------------------------------------------------------------------------------------------------------------------------------------------------------------------------------------------------------------------------------------------------------------------------------ACACAGTAGGTGATGCATCTGATTCTCCTACATCAGCTCCACGAATACCATCAC----------------------------------------------**

**SRR184103.sra.1382469 ------------------------------------------------TTCAGAGACAGGGCCAGCATACGAGGAATAAGTGAACAGTTGCTGGCACTCA-----------------------------------------------------------------------------------------------------------------------------------------------------------------------------------------------------------------------------------------------------------------------------------ACACAGTAG-------------------------------------------------------------------------------------------**

**SRR184103.sra.6102900 --------------------------------------------------------CAGGGCCAGCATACGAGGAATAAGTGAACAGTTGCTGGCACTCA-----------------------------------------------------------------------------------------------------------------------------------------------------------------------------------------------------------------------------------------------------------------------------------ACACAGTAGGTGATGCA-----------------------------------------------------------------------------------**

**SRR184103.sra.1830267 ---------------------------------------------------------------------------ATAAGTGAACAGTTGCTGGCACTCA-----------------------------------------------------------------------------------------------------------------------------------------------------------------------------------------------------------------------------------------------------------------------------------ACATAGTAGGTGATGCATCTGATTCTCCTACATCAG----------------------------------------------------------------**

**SRR184103.sra.4682958 ----------------------------------------------------------------------------TAAGTGAACAGTTGCTGGCACTCA-----------------------------------------------------------------------------------------------------------------------------------------------------------------------------------------------------------------------------------------------------------------------------------ACATAGTAGGTGATGCATCTGATTCTCCTACATCAGC---------------------------------------------------------------**

**SRR184103.sra.3413343 --------------------------------------------------------------------------------------GTTGCTGGCACTCA-----------------------------------------------------------------------------------------------------------------------------------------------------------------------------------------------------------------------------------------------------------------------------------ACATAGTAGGTGCTGCATCTGATTCTCCTACATCAGCTCCACGAATA-----------------------------------------------------**

**SRR184103.sra.4259096 ------------------------------------------------------------------------------------------CTGGCACTCA-----------------------------------------------------------------------------------------------------------------------------------------------------------------------------------------------------------------------------------------------------------------------------------ACATAGTAGGTGATGCATCTGATTCTCCTACATCAGCTCCACGAATACCAT-------------------------------------------------**

**SRR184103.sra.1715304 ----------------------------------------------------------------------------------------------CACTCA-----------------------------------------------------------------------------------------------------------------------------------------------------------------------------------------------------------------------------------------------------------------------------------ACACAGTAGGTGATGCATCTGATTCTCCTACATCAGCTCCACGAATACCATCACC---------------------------------------------**

**SRR184104.sra.1668473 ----------------------------------------------------------------GCACACGAGGAATAAGTGAACAGTTGCTGGCACTCA-----------------------------------------------------------------------------------------------------------------------------------------------------------------------------------------------------------------------------------------------------------------------------------ACATAGTAGGTGATGCATCTGATTC---------------------------------------------------------------------------**

**SRR184104.sra.846327 ---------------------------------------------------------------------------------GAACAGTTGCTGGCACTCA-----------------------------------------------------------------------------------------------------------------------------------------------------------------------------------------------------------------------------------------------------------------------------------ACATAGTAGGTGATGCCTCTGATTCTCCTACATCAGCTCCAC----------------------------------------------------------**

**SRR184104.sra.2287645 -------------------------------------------------------------------------------------AGTTGCTGGCACTCA-----------------------------------------------------------------------------------------------------------------------------------------------------------------------------------------------------------------------------------------------------------------------------------ACACAGTAGGTGATGCATCTGATTCTCCTACATCAGCTCCACGAAT------------------------------------------------------**

**SRR184104.sra.9739345 -------------------------------------------------------------------------------------AGTTGCTGGCACTCA-----------------------------------------------------------------------------------------------------------------------------------------------------------------------------------------------------------------------------------------------------------------------------------ACACAGTAGGTGATGCATCTGATTCTCCTACATCAGCTCCACGAAT------------------------------------------------------**

**SRR184104.sra.3992658 --------------------------------------------------------------------------------------GTTGCTGGCACTCA-----------------------------------------------------------------------------------------------------------------------------------------------------------------------------------------------------------------------------------------------------------------------------------ACATAGTAGGTGATGCATCTGATTCTCCTACATCAGCTCCACGAATA-----------------------------------------------------**

**SRR864485.sra.255227 --------------------------------------------------------------CAGCATACGAGGAATAAGTGAACAGTTGCTGGCACTCA-----------------------------------------------------------------------------------------------------------------------------------------------------------------------------------------------------------------------------------------------------------------------------------ACACAGTAGGTG----------------------------------------------------------------------------------------**

**SRR864485.sra.9746186 -------------------------------------------------------------------------GAATAAGTGAACAGTTGCTGGCACTCA-----------------------------------------------------------------------------------------------------------------------------------------------------------------------------------------------------------------------------------------------------------------------------------ACACAGTAGGTGATGCATCTGAT-----------------------------------------------------------------------------**

**SRR864485.sra.10732548 -------------------------------------------------------------------------GAATAAGTGAACAGTTGCTGGCACTCA-----------------------------------------------------------------------------------------------------------------------------------------------------------------------------------------------------------------------------------------------------------------------------------ACACAGTAGGTGATGCATCTGAT-----------------------------------------------------------------------------**

**SRR864485.sra.1166735 ----------------------------------------------------------------------------TAAGTGAACAGTTGCTGGCACTCA-----------------------------------------------------------------------------------------------------------------------------------------------------------------------------------------------------------------------------------------------------------------------------------ACCTAGTAGGTGATGCATCTGATTCT--------------------------------------------------------------------------**

**SRR865383.sra.4448107 -------------------------------------------------------------CCAGCACACGAGGAATAAGTGAACAGTTGCTGGCACTCA-----------------------------------------------------------------------------------------------------------------------------------------------------------------------------------------------------------------------------------------------------------------------------------ACATAGTAGGT-----------------------------------------------------------------------------------------**

**SRR865383.sra.10945444 -----------------------------------------------------------------CATACGAGGAATAAGTGAACAGTTGCTGGCACTCA-----------------------------------------------------------------------------------------------------------------------------------------------------------------------------------------------------------------------------------------------------------------------------------ACACAGTAGGTGATG-------------------------------------------------------------------------------------**

**SRR865383.sra.9967879 ----------------------------------------------------------------------GAGGAATAAGTGAACAGTTGCTGGCACTCA-----------------------------------------------------------------------------------------------------------------------------------------------------------------------------------------------------------------------------------------------------------------------------------ACACAGTAGGTGATGCATCT--------------------------------------------------------------------------------**

**SRR865383.sra.11281531 ------------------------------------------------------------------------------AGTGAACAGTTGCTGGCACTCA-----------------------------------------------------------------------------------------------------------------------------------------------------------------------------------------------------------------------------------------------------------------------------------ACATAGTAGGTGATGCATCTGATTCTCC------------------------------------------------------------------------**

**SRR865383.sra.1626748 ------------------------------------------------------------------------------------------CTGGCACTCA-----------------------------------------------------------------------------------------------------------------------------------------------------------------------------------------------------------------------------------------------------------------------------------ACACAGTAGGTGATGCATCTGATTCTCCTACATCAGCTCC------------------------------------------------------------**

**SRR865383.sra.8166763 -------------------------------------------------------------------------------------------TGGCACTCA-----------------------------------------------------------------------------------------------------------------------------------------------------------------------------------------------------------------------------------------------------------------------------------ACATAGTAGGTGATGCATCTGATTCTCCTACATCAGCTCCA-----------------------------------------------------------**

**SRR865383.sra.3724545 ---------------------------------------------------------------------------------------------GCACTCA-----------------------------------------------------------------------------------------------------------------------------------------------------------------------------------------------------------------------------------------------------------------------------------ACACAGTAGGTGATGCATCTGATTCTCCTACATCAGCTCCACG---------------------------------------------------------**

**SRR865902.sra.13126678 -------------------------------------------------------------------------------GTGAACAGTTGCTGGCACTCA-----------------------------------------------------------------------------------------------------------------------------------------------------------------------------------------------------------------------------------------------------------------------------------ACATAGTAGGTGATGCATCTGATTCTCCT-----------------------------------------------------------------------**

**SRR865902.sra.1390782 --------------------------------------------------------------------------------TGAACAGTTGCTGGCACTCA-----------------------------------------------------------------------------------------------------------------------------------------------------------------------------------------------------------------------------------------------------------------------------------ACATAGTAGGTGATGCATCTGATTCTCCTA----------------------------------------------------------------------**

**SRR866226.sra.5606856 ---------------------------------------------------------------------CGAGGAATAAGTGAACAGTTGCTGGCACTCA-----------------------------------------------------------------------------------------------------------------------------------------------------------------------------------------------------------------------------------------------------------------------------------ACATAGTAGGTGATGCATC---------------------------------------------------------------------------------**

**SRR866237.sra.9421602 -------------------------------------------------------------CCAGCACACGAGGAATAAGTGAACAGTTGCTGGCACTCA-----------------------------------------------------------------------------------------------------------------------------------------------------------------------------------------------------------------------------------------------------------------------------------ACATAGTAGGT-----------------------------------------------------------------------------------------**

**SRR866237.sra.20286508 ------------------------------------------------------------------------------------------CTGGCACTCA-----------------------------------------------------------------------------------------------------------------------------------------------------------------------------------------------------------------------------------------------------------------------------------ACACAGTAGGTGATGCATCTGATTCTCCTACATCAGCTCC------------------------------------------------------------**

**SRR866242.sra.10759806 --------------------------------------------------------------CAGCACACGAGGAATAAGTGAACAGTTGCTGGCACTCA-----------------------------------------------------------------------------------------------------------------------------------------------------------------------------------------------------------------------------------------------------------------------------------ACATAGTAGGTG----------------------------------------------------------------------------------------**

**SRR866242.sra.2770030 --------------------------------------------------------------------ACGAGGAATAAGTGAACAGTTGCTGGCACTCA-----------------------------------------------------------------------------------------------------------------------------------------------------------------------------------------------------------------------------------------------------------------------------------ACATAGTAGGTGATGCAT----------------------------------------------------------------------------------**

**SRR866242.sra.1195674 ---------------------------------------------------------------------CGAGGAATAAGTGAACAGTTGCTGGCACTCA-----------------------------------------------------------------------------------------------------------------------------------------------------------------------------------------------------------------------------------------------------------------------------------ACACAGTAGGTGATGCATC---------------------------------------------------------------------------------**

**SRR866242.sra.13424756 ------------------------------------------------------------------------GGAATAAGTGAACAGTTGCTGGCACTCA-----------------------------------------------------------------------------------------------------------------------------------------------------------------------------------------------------------------------------------------------------------------------------------ACATAGTAGGTGATGCATCTGA------------------------------------------------------------------------------**

**SRR866242.sra.256842 -------------------------------------------------------------------------GAATAAGTGAACAGTTGCTGGCACTCA-----------------------------------------------------------------------------------------------------------------------------------------------------------------------------------------------------------------------------------------------------------------------------------ACATAGTAGGTGATGCATCTGAT-----------------------------------------------------------------------------**

**SRR866242.sra.2181986 -------------------------------------------------------------------------GAATAAGTGAACAGTTGCTGGCACTCA-----------------------------------------------------------------------------------------------------------------------------------------------------------------------------------------------------------------------------------------------------------------------------------ACACAGTAGGTGATGCATCTGAT-----------------------------------------------------------------------------**

**SRR866242.sra.3990289 -------------------------------------------------------------------------------GTGAACAGTTGCTGGCACTCA-----------------------------------------------------------------------------------------------------------------------------------------------------------------------------------------------------------------------------------------------------------------------------------ACACAGTAGGTGATGCATCTGATTCTCCT-----------------------------------------------------------------------**

**SRR866243.sra.14812011 --------------------------------------------------------------------------------TGAACAGTTGCTGGCACTCA-----------------------------------------------------------------------------------------------------------------------------------------------------------------------------------------------------------------------------------------------------------------------------------ACATAGTAGGTGATGCATCTGATTCTCCTA----------------------------------------------------------------------**

**SRR866243.sra.9153005 --------------------------------------------------------------------------------------GTTGCTGGCACTCA-----------------------------------------------------------------------------------------------------------------------------------------------------------------------------------------------------------------------------------------------------------------------------------ACATAGTAGGTGATGCATCTGATTCTCCTACATCAG----------------------------------------------------------------**

**SRR866243.sra.2354693 ------------------------------------------------------------------------------------------CTGGCACTCA-----------------------------------------------------------------------------------------------------------------------------------------------------------------------------------------------------------------------------------------------------------------------------------ACACAGTAGGTGATGCATCTGATTCTCCTACATCAGCTCC------------------------------------------------------------**

**SRR866250.sra.6728833 ------------------------------------------------------------GCCAGCACACGAGGAATAAGTGAACAGTTGCTGGCACTCA-----------------------------------------------------------------------------------------------------------------------------------------------------------------------------------------------------------------------------------------------------------------------------------ACATAGTAGG------------------------------------------------------------------------------------------**

**SRR866250.sra.4991040 ---------------------------------------------------------------------CGAGGAATAAGTGAACAGTTGCTGGCACTCA-----------------------------------------------------------------------------------------------------------------------------------------------------------------------------------------------------------------------------------------------------------------------------------ACATAGTAGGTGATGCATC---------------------------------------------------------------------------------**

**SRR866250.sra.5005162 ---------------------------------------------------------------------CGAGGAATAAGTGAACAGTTGCTGGCACTCA-----------------------------------------------------------------------------------------------------------------------------------------------------------------------------------------------------------------------------------------------------------------------------------ACATAGTAGGTGATGCATC---------------------------------------------------------------------------------**

**SRR866250.sra.7677174 ---------------------------------------------------------------------CGAGGAATAAGTGAACAGTTGCTGGCACTCA-----------------------------------------------------------------------------------------------------------------------------------------------------------------------------------------------------------------------------------------------------------------------------------ACACAGTAGGTGATGCATC---------------------------------------------------------------------------------**

**SRR866250.sra.10734484 -----------------------------------------------------------------------AGGAATAAGTGAACAGTTGCTGGCACTCA-----------------------------------------------------------------------------------------------------------------------------------------------------------------------------------------------------------------------------------------------------------------------------------ACATAGTAGGTGATGCATCTG-------------------------------------------------------------------------------**

**SRR866250.sra.16815557 -------------------------------------------------------------------------GAATAAGTGAACAGTTGCTGGCACTCA-----------------------------------------------------------------------------------------------------------------------------------------------------------------------------------------------------------------------------------------------------------------------------------ACATAGTAGGTGATGCATCTGAT-----------------------------------------------------------------------------**

**SRR866266.sra.3738582 --------------------------------------------------------------CAGCATACGAGGAATAAGTGAACAGTTGCTGGCACTCA-----------------------------------------------------------------------------------------------------------------------------------------------------------------------------------------------------------------------------------------------------------------------------------ACACAGTAGGTG----------------------------------------------------------------------------------------**

**SRR866266.sra.10394873 --------------------------------------------------------------CAGCACACGAGGAATAAGTGAACAGTTGCTGGCACTCA-----------------------------------------------------------------------------------------------------------------------------------------------------------------------------------------------------------------------------------------------------------------------------------ACATAGTAGGTG----------------------------------------------------------------------------------------**

**SRR866266.sra.15967051 -------------------------------------------------------------------CACGAGGAATAAGTGAACAGTTGCTGGCACTCA-----------------------------------------------------------------------------------------------------------------------------------------------------------------------------------------------------------------------------------------------------------------------------------ACATAGTAGGTGATGCA-----------------------------------------------------------------------------------**

**SRR866266.sra.61808 ---------------------------------------------------------------------CGAGGAATAAGTGAACAGTTGCTGGCACTCA-----------------------------------------------------------------------------------------------------------------------------------------------------------------------------------------------------------------------------------------------------------------------------------ACATAGTAGGTGATGCATC---------------------------------------------------------------------------------**

**SRR866266.sra.4230581 ----------------------------------------------------------------------------TAAGTGAACAGTTGCTGGCACTCA-----------------------------------------------------------------------------------------------------------------------------------------------------------------------------------------------------------------------------------------------------------------------------------ACACAGTAGGTGATGCATCTGATTCT--------------------------------------------------------------------------**





Figure S3. WGS reads mapped crossing Site 1. The exact nucleotide sequences in this figure are visible when it is viewed at the 250% zoom setting.

**↓**

***Site1*** ataagagtatctgaagatgaaaatgtccttacaataggtaatatctaaagaataattcccgtattgtttattcacatataaattgagtatatatctggacattagtttaggcttaatgaagaacttgttcaaattttttattggttgcgatctcttcttccttttttttgcatatttacaactctacatgtaaactatgt

***SRR307668.sra.20911835*** -------------------------------------------------------------TATTGTTTATTCACATATAAATTGAGTATATATCTGGACATTAGTTTAGGCTTAATGAAGAACTTGT------------------------------------------------------------------------

***SRR307703.sra.11867961*** --------------------------------------------------------------------TATTCACATATAAATTGAGTATATATCTGGACATTAGTTTAGGCTTAATGAAGAACTTGTTCA---------------------------------------------------------------------

***SRR307703.sra.6842705***  ---------------------------------------------------------------------ATTCACATATAAATTGAGTATATATCTGGACATTAGTTTAGGCTTAATGAAGAACTTGTTCAAATTT----------------------------------------------------------------

***SRR307686.sra.8916980***  ---------------------------------------------------------------------ATTCACATATAAATTGAGTATATATCTGGACATTAGTTTAGGCTTAATGAAGAACTTG-------------------------------------------------------------------------

***SRR307686.sra.19641854*** ------------------------------------------------------------------------------TAAATTGAGTATATATCTGGACATTAGTTTAGGCTTAATGAAGAACTTGCTCAAATTTTTTA------------------------------------------------------------

***SRR307691.sra.14196321*** ------------------------------------------------------------------------CACATATAAATTGAGTATATATCTGGACATTAGTTTAGGCTTAATGAAGAA-----------------------------------------------------------------------------

***SRR307691.sra.5757195***  --------------------------------------------------------------------------------AATTGAGTATATATCTGGACATTAGTTTAGGCTTAATGAAGAACTTGTTCAAATTTTTTAGC----------------------------------------------------------

***SRR307691.sra.2382222***  --------------------------------------------------------------------------------CTTTGAGTATATATCTGGACATTAGTTTAGGCTTAATGAAGAACTTGTTCAAATTTTTTATT----------------------------------------------------------

***SRR307691.sra.5228627***  ----------------------------------------------------------------------------------------ATATATCTGGACATTAGTTTAGGCTTAATGAAGAACTTGTTCAAATTTTTTATTGGTTGCCA--------------------------------------------------

***SRR307691.sra.19912973*** -----------------------------------------------------------------------------------------TATATCTGGACATTAGTTTAGGCTTAATGAAGAACTTGTTCAAATTTTTTATTGGTTGCGAC-------------------------------------------------

***SRR307611.sra.16527052*** ---------------------------------------------------------------------------------------------TCTGGACATTAGTTTAGGCTTAATGAAGAACTTGTTCAAATTTTTTATTGGTTGCGATCT-----------------------------------------------

***SRR307666.sra.15423643*** ---------------------------------------------------------------------------------ATTGAGTATATATCTGGACATTAGTTTAGGCTTAATGAAGAACTTGTTCAAATTTTTTAT-----------------------------------------------------------

***SRR307648.sra.15860875*** --------------------------------------------------------------------TATTCACATATAANTTGAGTATATATCTGGACATTAGTTTAGGCTTAATGAAGAACTTGTTCA---------------------------------------------------------------------

***SRR307648.sra.14591446*** ---------------------------------------------------------------------ATTCACATATAAATTGAGTATATATCTGGACATTAGTTTAGGCTTAATGAAGAACTTGTTCAA--------------------------------------------------------------------

***SRR307701.sra.46025***  -------------------------------------------------GAATAATTCCCGTATTGTTTATTCACATATAAATTGAGTATATATCTGGACATTAGTTTAGGCTTAA------------------------------------------------------------------------------------

***SRR307701.sra.2514979***  ----------------------------------------------------------------------------------------ATATATCTGGACATTAGTTTAGGCTTAATGAAGAACTTGTTCAAATTTTTTATNGGTTGCGATC------------------------------------------------

***SRR307701.sra.17955035*** ---------------------------------------------------------------------------------------------TCTGGACATTAGTTTAGGCTTAATGAAGAACTTGTTCAAATTTTTTATTGGTTGCGATCTCTTC-------------------------------------------

Figure S4. WGS reads mapped crossing Site 2. The exact nucleotide sequences in this figure are visible when it is viewed at the 250% zoom setting.

**↓**

***Site2***  GCTTTCAAAGAGATCATTACGATCCTAGGCCATCTACATTCAGAGACAGGGCCAGCACACGAGGAATAAGTGAACAGTTGCTGGCACTCAgtaagcttgaattagtttaggcttaatgaagaacttgttcaatttttttattggttgcgatctcttcttcctttttttgcatatttacaactctacatgtaaactatgtt

***SRR307668.sra.2540821***  --------------------------------------TTCAGAGACAGGGCCAGCACACGAGGAATAAGTGAACAGTTGCTGGCACTCAGTAAGCTTGAATTAG-----------------------------------------------------------------------------------------------

***SRR307668.sra.10052925*** --------------------------------------------------------ACACGAGGAATAAGTGAACAGTTGCTGGCACTCAGTAAGCTTGAATTAGTTTAGGNTTAATGAAG-------------------------------------------------------------------------------

***SRR307703.sra.18572963*** -----------------------------------------------------------------------------------GCACTCAGTAAGCTTGAATTAGTTTAGGCTTAATGAAGAACTTGTTCAATTTTTTTATTGGTT------------------------------------------------------

***SRR307686.sra.6953648***  ----------------------------------------------------------------------------------------------GCTTGAATTAGTTTAGGCTTAATGAAGAACTTGTTCAATTTTTTTATTGGTTGCGATCTCTT--------------------------------------------

***SRR307691.sra.15230150*** ---------------------------------------------ACAGGGCCAGCACACGAGGAATAAGTGAACAGTTGCTGGCACTCAGTAAGCTTGAATTAGAT---------------------------------------------------------------------------------------------

***SRR307691.sra.10162142*** -----------------------------------------------------------------------GAACAGTTGCTGGCACTCAGTAAGCTTGAATTAGTTTAGGCTTAATGAAGAACTTGTTCATT-------------------------------------------------------------------

***SRR307691.sra.14158436*** --------------------------------------------------------------------------CAGTTGCTGGCACTCAGTAAGCTTGAATTAGTTTAGGCTTAATGAAGAACTTGTTCAATTGC----------------------------------------------------------------

***SRR307611.sra.11941603*** ------------------------------------------------GGGCCAGCACACGAGGAATAAGTGAACAGTTGCTGGCACTCAGTAAGCTTGAATTAGTTT--------------------------------------------------------------------------------------------

***SRR307611.sra.1524825***  ----------------------------------------------------------ACGAGGAATAAGNGAACAGTTGCTGGCACTCAGTAAGCTTGAATTAGTTTAGGCTTAATG----------------------------------------------------------------------------------

***SRR307611.sra.11402320*** ------------------------------------------------------------GAGGAATAAGTGAACAGTTGCTGGCACTCAGTAAGCTTGAATTAGTTTAGGCTTAATGAA--------------------------------------------------------------------------------

***SRR307666.sra.4177666***  -------------------------------------------------GGCCAGCACACGAGGAATAAGTGAACAGTTGCTGGCACTCAGTAAGCTTGAATTAGTTTA-------------------------------------------------------------------------------------------

***SRR307666.sra.14915312*** --------------------------------------------------GCCAGCACACGAGGAATAAGTGAACAGTTGCTCGCACTCAGTAAGCTTGAATTAGTTTAG------------------------------------------------------------------------------------------

***SRR307666.sra.4096851***  -----------------------------------------------------------------------------------GCACTCAGTAAGCTTGAATTAGTTTAGGCTTAATGAAGAACTTGTTCAATTTTTTTATTG---------------------------------------------------------

***SRR307648.sra.15049691*** ----------------------------------------------------------------------TGAACAGTTGCTGGCACTCAGTAAGCTTGAATTAGTTTAGGCTTAATGAAGAACTTGTTCAAT-------------------------------------------------------------------

***SRR307648.sra.2722934***  -----------------------------------------------------------------------GAACAGTTGCTGGCACTCAGTAAGCTTGAATTAGTTTAGGCTTAATGAAGAACTTGTTCAATTTTT---------------------------------------------------------------

***SRR307648.sra.18667884*** -----------------------------------------------------------------------------------------AGTAAGCTTGAATTAGTTTAGGCTTAATGAAGAACTTGTTCAATTTTTTTATTGGTTGCGATCTCT---------------------------------------------

***SRR307701.sra.6383738***  ----------------------------------------------------------ACGAGGAATAAGTGAACAGTTGCTGGCACTCAGTAAGCTTGAATTAGTTTAGGCTTAATGAAGAACT---------------------------------------------------------------------------

***SRR307701.sra.18876344*** ------------------------------------------------------------GAGGAATAAGTGAACAGTTGCTGGCACTCAGTAAGCTTGAATTAGTTTAGGCTTAATGAAGAAC----------------------------------------------------------------------------

Figure S5. WGS reads mapped crossing Site 3. The exact nucleotide sequences in this figure are visible when it is viewed at the 250% zoom setting.

**↓**

***Site3***  GCTTTCAAAGAGATCATTACGATCCTAGGCCATCTACATTCAGAGACAGGGCCAGCACACGAGGAATAAGTGAGCAGTTACTGGCACTCAGTAAGCTTGAATTCAGGAAATTCTTTTTGATTCTAAACTACATTGGGAGgtaaatttagcatttattcaaatatttactttgtgacttctatattgctacaagttggtat

***SRR307611.sra.17511416*** --------------------------------------------------------ACACGAGGAATAAGTGAGCAGTTACTGGCACTCAGTAAGCTTGAATTCAGGAAAGCCTTTT-----------------------------------------------------------------------------------

***SRR307611.sra.1836556***  ---------------------------------------------------------------------------AGTTACTGGCACTCAGTAAGCTTGTATTCAGGAAATTCTTTTTGATTCTAAACTACATTGGGAGGTAAA--------------------------------------------------------

***SRR307611.sra.337437***  ------------------------------------------------------------------------------------CACTCAGTAAGCTTGAATTCAGGAAATTCTTTTTGATTCTAAACTACATTGGGAGGTAAAT-------------------------------------------------------

***SRR307611.sra.7788356***  ----------------------------------------------------------------------------------------CAGTAAGCTTGAATTCAGNAAATTCTTTTTGATTCTAAACTACATTGGGAGGTAAATTTAGCATTTATT-------------------------------------------

***SRR307611.sra.12059209*** -----------------------------------------------------------------------------------------AGTAAGCTTGAATTCAGGAAATTCTTTTTGATTCTAAACTACATTGGGAGGTAAATTTAGC--------------------------------------------------

***SRR307648.sra.19729397*** ---------------------------------------------------CCAGCACACGAGGAATAAGTGAGCAGTTACTGGCACTCAGTAAGCTTGAATTCAGGAAATTCTTTT-----------------------------------------------------------------------------------

***SRR307648.sra.2812439***  ----------------------------------------------------------------------------------------------GCTTGAATTCAGGAAATTCTTTTTGATTCTAAACTACATTGGGAGGTAAATTTAGCATTTATT-------------------------------------------

***SRR307666.sra.7935592***  -----------------------------------------------GGGGCCAGCACACGAGGAATAAGTGAGCAGTTACTGGCACTCAGTAAGCTTGAATTCAGGAAA------------------------------------------------------------------------------------------

***SRR307666.sra.2487878***  ----------------------------------------------------CAGCACACGAGGAATAAGTGAGCAGTTACTGGCACTCAGTAAGCTTGAATTCAGGAAATTCTT-------------------------------------------------------------------------------------

***SRR307666.sra.9541505***  ----------------------------------------------------------------------------------------CAGTAAGCTTGAATTCAGGAAATTCTTTTTGATTCTAAACTACATTGGGAGGTAAATTTAGCATTT----------------------------------------------

***SRR307668.sra.15396543*** -----------------------------------------AGAGACAGGGCCAGCACACGAGGAATAAGTGAGCAGTTACTGGCACTCAGTAAGCTTGAATTCAGGA--------------------------------------------------------------------------------------------

***SRR307668.sra.18852112*** -------------------------------------------------------------------AAGTGAGCAGTTACTGGCACTCAGTAAGCTTGAATTCAGGAAATTCTTTTTGATTCTAAACTACA--------------------------------------------------------------------

***SRR307668.sra.14684307*** ---------------------------------------------------------------------------AGTTACTGGCACTCAGTAAGCTTGAATTCAGGAAATTCTTTTTGATTCTAAACTACATTGGGAGGTA----------------------------------------------------------

***SRR307668.sra.874384***  ---------------------------------------------------------------------------------TGGCACTCAGTAAGCTTGAATTCAGGAAATTCTTTTTGATTCTAAACTACATTGNGAGGTAAATTTA----------------------------------------------------

***SRR307686.sra.18388025*** --------------------------------------------------------------------------CAGTTACTGGCACTCAGTAAGCTTGAATTCAGGAAATTCTTTTTGATTCTAAACTACA--------------------------------------------------------------------

***SRR307691.sra.2581471***  ------------------------------------------------------------------TAAGTGAGCAGTTACTGGCACTCAGTAAGCTTGAATTCAGGAAATTCTTTTTGATTCTAAAA------------------------------------------------------------------------

***SRR307703.sra.17318168*** -------------------------------------------------------CACACGAGGAATAAGCGAGCAGTTACTGGCACTCAGTAAGCTTGAATTCAGGAATNTCTTTTT----------------------------------------------------------------------------------

***SRR307703.sra.13140955*** ------------------------------------------------------------------TAAGTGAGCAGTTACTGGCACTCAGTAAGCTTGAATTCAGGAAATTCTTTTTGATTCTAAACTACAT-------------------------------------------------------------------
