# Supplementary material for: Intron gain by tandem genomic duplication: a novel case in a potato gene encoding RNA-dependent RNA polymerase
Source: PeerJ. 2016 Jul 26;4:e2272. doi: 10.7717/peerj.2272 (PMC4974935; doi:10.7717/peerj.2272)
Supplement: Supplemental Information 3 [file peerj-04-2272-s003.docx]

Supplemental Information 3 of

**Intron gain by tandem genomic duplication: a novel case in a potato gene encoding RNA-dependent RNA polymerase**

Ming-Yue Ma, Xin-Ran Lan and Deng-Ke Niu

Table S1. The RNA-Seq reads mapped crossing the exon-exon boundary of the target intron.

| Tissue | Read IDs | Read Sequences |
| --- | --- | --- |
| Leaf | ERR305631.sra.5633486 | CATCTACATTCAGAGACAGGGCCAGCATACGAGGAATAAGTGAACAGTTGCTGGCACTCAACACAGTAGGTGATGCATCTGATTCTCCTA |
| Leaf | ERR305631.sra.14764201 | ATCTACATTCAGAGACAGGGCCAGCACACGAGGAATAAGTGAACAGTTGCTGGCACTCAACATAGTAGGTGATGCATCTGATTCTCCTAC |
| Leaf | ERR305631.sra.1954676 | ATTCAGAGACAGGGCCAGCACACGAGGAATAAGTGAACAGTTGCTGGCACTCAACATAGTAGGTGATGCATCTGATTCTCCTACATCAGC |
| Leaf | ERR305631.sra.25528009 | ATTCAGAGACAGGGCCAGCACACGAGGAATAAGTGAACAGTTGCTGGCACTCAACATAGTAGGTGATGCATCTGATTCTCCTACATCAGC |
| Leaf | ERR305631.sra.11034325 | AGACAGGGCCAGCATACGAGGAATAAGTGAACAGTTGCTGGCACTCAACACAGTAGGTGATGCATCTGATTCTCCTACATCAGCTCCACG |
| Leaf | ERR305631.sra.21174123 | GACAGGGCCAGCATACGAGGAATAAGTGAACAGTTGCTGGCACTCAACACAGTAGGTGATGCATCTGATTCTCCTACATCAGCTCCACGA |
| Leaf | ERR305631.sra.14962990 | GGGCCAGCATACGAGGAATAAGTGAACAGTTGCTGGCACTCAACACAGTAGGTGATGCATCTGATTCTCCTACATCAGCTCCACGAATAC |
| Leaf | ERR305631.sra.1208780 | GCCAGCATACGAGGAATAAGTGAACAGTTGCTGGCACTCAACACAGTAGGTGATGCATCTGATTCTCCTACATCAGCTCCACGAATACCA |
| Leaf | ERR305631.sra.19438135 | GTGAACAGTTGCTGGCACTCAACATAGTAGGTGATGCATCTGATTCTCCTACATCAGCTCCACGAATACCATCACCTCCAATGAGTCCAG |
| Leaf | ERR305631.sra.21551313 | AGTTGCTGGCACTCAACATAGTAGGTGATGCATCTGATTCTCCTACATCAGCTCCACGAATACCATCACCTCCAATGAGTCCAGTGACAA |
| Leaf | ERR305632.sra.5125036 | AAGAGATCATTACGATCCTAGGCCATCTACATTCAGAGACAGGGCCAGCACACGAGGAATAAGTGAACAGTTACTGGCACTCAACATAGT |
| Leaf | ERR305632.sra.17798760 | CGATCCTAGGCCATCTACATTCAGAGACAGGGCCAGCACACGAGGAATAAGTGAACAGTTGCTGGCACTCAACATAGTAGGTGATGCATC |
| Leaf | ERR305632.sra.19889662 | AGGCCATCTACATTCAGAGACAGGGCCAGCACACGAGGAATAAGTGAACAGTTGCTGGCACTCAACATAGTAGGTGATGCATCTGATTCT |
| Leaf | ERR305632.sra.23851887 | AGGCCATCTACATTCAGAGACAGGGCCAGCACACGAGGAATAAGTGAACAGTTGCTCGCACTCAACATAGTAGGTGATGCATCTGATTCT |
| Leaf | ERR305632.sra.22237812 | GGCCATCTACATTCAGAGACAGGGCCAGCACACGAGGAATAAGTGAACAGTTGCTGGCACTCAACATAGTAGGTGATGCATCTGATTCTC |
| Leaf | ERR305632.sra.17496368 | GCCATCTACATTCAGAGACAGGGCCAGCACACGAGGAATAAGTGAACAGTTGCTGGCACTCAACATAGTAGGTGATGCATCTGATTCTCC |
| Leaf | ERR305632.sra.20019441 | CTGGCACTCAACATAGTAGGTGATGCATCTGATTCTCCTACATCAGCTCCACGAATACCATCACCTCCAATGAGTCCAGTGACAACTAGC |
| Leaf | SRR1207283.sra.9962581 | TACGATCCTAGGCCATCTACATTCAGAGACAGGGCCAGCACACGAGGAATAAGAGAACAGTTGCTGGCACTCAACATAGTAGGTGATGCA |
| Leaf | SRR1207283.sra.23466160 | CGATCCTAGGCCATCTACATTCAGAGACAGGGCCAGCACACGAGGAATAAGTGAACAGTTGCTGGCACTCAACATAGTAGGTGATGCATC |
| Leaf | SRR1207283.sra.21759433 | CTAGGCCATCTACATTCAGAGACAGGGCCAGCACACGAGGAATAAGTGAACAGTTGCTGGCACTCAACATAGTAGGTGATGCATCTGATT |
| Leaf | SRR1207283.sra.22329356 | CTAGGCCATCTACATTCAGAGACAGGGCCAGCACACGAGGAATAAGTGAACAGTTGCTGGCACTCAACATAGTAGGTGATGCATCTGATT |
| Leaf | SRR1207283.sra.5226164 | GGCCATCTACATTCAGAGACAGGGCCAGCATACGAGGAATAAGTGAACAGTTGCTGGCACTCAACACAGTAGGTGATGCATCTGATTCTC |
| Leaf | SRR1207283.sra.22364697 | GCCATCTACATTCAGAGACAGGGCCAGCACACGAGGAATAAGTGAACAGTTGCTGGCACTCAACATAGTAGGTGATGCATCTGATTCTCC |
| Leaf | SRR1207283.sra.24433899 | GCCATCTACATTCAGAGACAGGGCCAGCACACGAGGAATAAGTGAACAGTTGCTGGCACTCAACATAGTAGGTGATGCATCTGATTCTCC |
| Leaf | SRR1207283.sra.22477210 | CAGAGACAGGGCCAGCATACGAGGAATAAGTGAACAGTTGCTGGCACTCAACACAGTAGGTGATGCATCTGATTCTCCTACATCAGCTCC |
| Leaf | SRR1207283.sra.3800679 | AGAGACAGGGCCAGCACACGAGGAATAAGTGAACAGTTGCTGGCACTCAACATAGTAGGTGATGCATCTGATTCTCCTACATCAGCTCCA |
| Leaf | SRR1207283.sra.5373704 | GAGACAGGGCCAGCACACGAGGAATAAGTGAACAGTTGCTGGCACTCAACATAGTAGGTGATGCATCTGATTCTCCTACATCAGCTCCAC |
| Leaf | SRR1207283.sra.4383324 | AGACAGGGCCAGCACACGAGGAATAAGTGAACAGTTGCTGGCACTCAACATAGTAGGTGATGCATCTGATTCTCCTACATCAGCTCCACG |
| Leaf | SRR1207283.sra.18821448 | AGACAGGGCCAGCACACGAGGAATAAGTGAACAGTTGCTGGCACTCAACATAGTAGGTGATGCATCTGATTCTCCTACATCAGCTCCACG |
| Leaf | SRR1207283.sra.19790545 | GTTGCTGGCACTCAACATAGTAGGTGATGCATCTGATTCTCCTACATCAGCTCCACGAATACCATCACCTCCAATGAGTCCAGTGACAAC |
| Leaf | SRR1207283.sra.15936888 | CTGGCACTCAACATAGTAGGTGATGCATCTGATTCTCCTACATCAGCTCCACGAATACCATCACCTCCAATGAGTCCAGTGACAACTAGC |
| Leaf | SRR1207284.sra.6983677 | CAAAGAGATCATTACGATCCTAGGCCATCTACATTCAGAGACAGGGCCAGCACACGAGGAATAAGTGAACAGTTGCTGGCACTCAACATA |
| Leaf | SRR1207284.sra.13221004 | CAAAGAGATCATTACGATCCTAGGCCATCTACATTCAGAGACAGGGCCAGCACACGAGGAATAAGTGAACAGTTGCTGGCACTCAACATA |
| Leaf | SRR1207284.sra.737394 | GAGATCATTACGATCCTAGGCCATCTACATTCAGAGACAGGGCCAGCACACGAGGAATAAGTGAACAGTTGCTGGCACTCAACATAGTAG |
| Leaf | SRR1207284.sra.8745191 | CATTACGATCCTAGGCCATCTACATTCAGAGACAGGGCCAGCACACGAGGAATAAGTGATCAGTTGCTGGCACTCAACATAGTAGGTGAT |
| Leaf | SRR1207284.sra.11182140 | ATTACGATCCTAGGCCATCTACATTCAGAGACAGGGCCAGCACACGAGGAATAAGTGAACAGTTGCTGGCACTCAACATAGTAGGTGATG |
| Leaf | SRR1207284.sra.15679219 | TTACGATCCTAGGCCATCTACATTCAGAGACAGGGCCAGCACACGAGGAATAAGTGAACAGTTGCTGGCACTCAACATAGTAGGTGATGC |
| Leaf | SRR1207284.sra.12578766 | TACGATCCTAGGCCATCTACATTCAGAGACAGGGCCAGCACACGAGGAATAAGTGAACAGTTGCTGGCACTCAACATAGTAGGTGATGCA |
| Leaf | SRR1207284.sra.3184424 | AGGCCATCTACATTCAGAGACAGGGCCAGCACACGAGGAATAAGTGAACAGTTGCTGGCACTCAACATAGTAGGTGATGCATCTGATTCT |
| Leaf | SRR1207284.sra.21163685 | AGGCCATCTACATTCAGAGACAGGGCCAGCACACGAGGAATAAGTGAATAGTTGCTGGCACTCAACATAGTAGGTGATGCATCTGATTCT |
| Leaf | SRR1207284.sra.12838668 | CATCTACATTCAGAGACAGGGCCAGCACACGAGGAATAAGTGAACAGTTGCTGGCACTCAACATAGTAGGTGATGCATCTGATTCTCCTA |
| Leaf | SRR1207284.sra.15602843 | TTCAGAGACAGGGCCAGCACACGAGGAATAAGTGAACAGTTGCTGGCACTCAACATAGTAGGTGATGCATCTGATTCTCCTACATCAGCT |
| Leaf | SRR1207284.sra.308417 | GAGACAGGGCCAGCACACGAGGAATAAGTGAACAGTTGCTGGCACTCAACATAGTAGGTGATGCATCTGATTCTCCTACATCAGCTCCAC |
| Leaf | SRR1207284.sra.20128638 | GCACACGAGGAATAAGTGAACAGTTGCTGGCACTCAACATAGTAGGTGATGCATCTGATTCTCCTACATCAGCTCCACGAATACCATCAC |
| Leaf | SRR1207284.sra.2009732 | AGTGAACAGTTGCTGGCACTCAACACAGTAGGTGATGCATCTGATTCTCCTACATCAGCTCCACGAATACCATCACATCCAATGAGTCCA |
| Leaf | SRR1207284.sra.7527762 | AGTGAACAGTTGCTGGCACTCAACACAGTAGGTGATGCATCTGATTCTCCTACATCAGCTCCACGAATACCATCACATCCAATGAGTCCA |
| Leaf | SRR1207285.sra.19310059 | GATCATTACGATCCTAGGCCATCTACATTCAGAGACAGGGCCAGCACACGAGGAATAAGTGAACAGTTGCTGGCACTCAACATAGTAGGT |
| Leaf | SRR1207285.sra.5814346 | TTACGATCCTAGGCCATCTACATTCAGAGACAGGGCCAGCACACGAGGAATAAGTGAAAAGTTGCTGGCACTCAACATAGTAGGTGATGC |
| Leaf | SRR1207285.sra.20492630 | CATTCAGAGACAGGGCCAGCACACGAGGAATAAGTGAACAGTTGCTGGCACTCAACATAGTAGGTGATGCATCTGATTCTCCTACATCAG |
| Leaf | SRR1207285.sra.15254802 | ATTCAGAGACAGGGCCAGCACACGAGGAATAAGTGAACAGTTGCTGGCACTCAACATAGTAGGTGATGCATCTGATTCTCCTACATCAGC |
| Leaf | SRR1207285.sra.9590300 | ATTCAGAGACAGGGCCAGCACACGAGGAATAAGTGAACAGTTGCTGGCACTCAACATAGTAGGTGATGCATCTGATTCTCCTACATCAGC |
| Leaf | SRR1207285.sra.20691870 | ATTCAGAGACAGGGCCAGCACACGAGGAATAAGTGAACAGTTGCTGGCACTCAACATAGTAGGTGATGCATCTGATTCTCCTACATCAGC |
| Leaf | SRR1207285.sra.18633958 | GAGACAGGGCCAGCATACGAGGAATAAGTGAACAGTTGCTGGCACTCAACACAGTAGGTGATGCATCTGATTCTCCTACATCAGCTCCAC |
| Leaf | SRR1207285.sra.22438572 | GACAGGGCCAGCACACGAGGAATAAGTGAACAGTTGCTGGCACTCAACATAGTAGGTGATGCATCTGATTCTCCTACATCAGCTCCACGG |
| Leaf | SRR1207285.sra.59217 | GGGCCAGCACACGAGGAATAAGTGAACAGTTGCTGGCACTCAACATAGTAGGTGATGCATCTGATTCTCCTACATCAGCTCCACGAATAC |
| Leaf | SRR1207285.sra.16151887 | GCATACGAGGAATAAGTGAACAGTTGCTGGCACTCAACACAGTAGGTGATGCATCTGATTCTCCTACATCAGCTCCACGAATACCATCAC |
| Leaf | SRR1207285.sra.21592470 | GCATACGAGGAATAAGTGAACAGTTGCTGGCACTCAACACAGTAGGTGATGCATCTGATTCTCCTACATCAGCTCCACGAATACCATCAC |
| Mixed Tissues | SRR184103.sra.1382469 | TTCAGAGACAGGGCCAGCATACGAGGAATAAGTGAACAGTTGCTGGCACTCAACACAGTAG |
| Mixed Tissues | SRR184103.sra.6102900 | CAGGGCCAGCATACGAGGAATAAGTGAACAGTTGCTGGCACTCAACACAGTAGGTGATGCA |
| Mixed Tissues | SRR184103.sra.1830267 | ATAAGTGAACAGTTGCTGGCACTCAACATAGTAGGTGATGCATCTGATTCTCCTACATCAG |
| Mixed Tissues | SRR184103.sra.4682958 | TAAGTGAACAGTTGCTGGCACTCAACATAGTAGGTGATGCATCTGATTCTCCTACATCAGC |
| Mixed Tissues | SRR184103.sra.3413343 | GTTGCTGGCACTCAACATAGTAGGTGCTGCATCTGATTCTCCTACATCAGCTCCACGAATA |
| Mixed Tissues | SRR184103.sra.4259096 | CTGGCACTCAACATAGTAGGTGATGCATCTGATTCTCCTACATCAGCTCCACGAATACCAT |
| Mixed Tissues | SRR184103.sra.1715304 | CACTCAACACAGTAGGTGATGCATCTGATTCTCCTACATCAGCTCCACGAATACCATCACC |
| Mixed Tissues | SRR184104.sra.1668473 | GCACACGAGGAATAAGTGAACAGTTGCTGGCACTCAACATAGTAGGTGATGCATCTGATTC |
| Mixed Tissues | SRR184104.sra.846327 | GAACAGTTGCTGGCACTCAACATAGTAGGTGATGCCTCTGATTCTCCTACATCAGCTCCAC |
| Mixed Tissues | SRR184104.sra.2287645 | AGTTGCTGGCACTCAACACAGTAGGTGATGCATCTGATTCTCCTACATCAGCTCCACGAAT |
| Mixed Tissues | SRR184104.sra.9739345 | AGTTGCTGGCACTCAACACAGTAGGTGATGCATCTGATTCTCCTACATCAGCTCCACGAAT |
| Mixed Tissues | SRR184104.sra.3992658 | GTTGCTGGCACTCAACATAGTAGGTGATGCATCTGATTCTCCTACATCAGCTCCACGAATA |
| Tuber | SRR864485.sra.255227 | CAGCATACGAGGAATAAGTGAACAGTTGCTGGCACTCAACACAGTAGGTG |
| Tuber | SRR864485.sra.9746186 | GAATAAGTGAACAGTTGCTGGCACTCAACACAGTAGGTGATGCATCTGAT |
| Tuber | SRR864485.sra.10732548 | GAATAAGTGAACAGTTGCTGGCACTCAACACAGTAGGTGATGCATCTGAT |
| Tuber | SRR864485.sra.1166735 | TAAGTGAACAGTTGCTGGCACTCAACCTAGTAGGTGATGCATCTGATTCT |
| Tuber | SRR865383.sra.4448107 | CCAGCACACGAGGAATAAGTGAACAGTTGCTGGCACTCAACATAGTAGGT |
| Tuber | SRR865383.sra.10945444 | CATACGAGGAATAAGTGAACAGTTGCTGGCACTCAACACAGTAGGTGATG |
| Tuber | SRR865383.sra.9967879 | GAGGAATAAGTGAACAGTTGCTGGCACTCAACACAGTAGGTGATGCATCT |
| Tuber | SRR865383.sra.11281531 | AGTGAACAGTTGCTGGCACTCAACATAGTAGGTGATGCATCTGATTCTCC |
| Tuber | SRR865383.sra.1626748 | CTGGCACTCAACACAGTAGGTGATGCATCTGATTCTCCTACATCAGCTCC |
| Tuber | SRR865383.sra.8166763 | TGGCACTCAACATAGTAGGTGATGCATCTGATTCTCCTACATCAGCTCCA |
| Tuber | SRR865383.sra.3724545 | GCACTCAACACAGTAGGTGATGCATCTGATTCTCCTACATCAGCTCCACG |
| Tuber | SRR865902.sra.13126678 | GTGAACAGTTGCTGGCACTCAACATAGTAGGTGATGCATCTGATTCTCCT |
| Tuber | SRR865902.sra.1390782 | TGAACAGTTGCTGGCACTCAACATAGTAGGTGATGCATCTGATTCTCCTA |
| Tuber | SRR866226.sra.5606856 | CGAGGAATAAGTGAACAGTTGCTGGCACTCAACATAGTAGGTGATGCATC |
| Tuber | SRR866237.sra.9421602 | CCAGCACACGAGGAATAAGTGAACAGTTGCTGGCACTCAACATAGTAGGT |
| Tuber | SRR866237.sra.20286508 | CTGGCACTCAACACAGTAGGTGATGCATCTGATTCTCCTACATCAGCTCC |
| Tuber | SRR866242.sra.10759806 | CAGCACACGAGGAATAAGTGAACAGTTGCTGGCACTCAACATAGTAGGTG |
| Tuber | SRR866242.sra.2770030 | ACGAGGAATAAGTGAACAGTTGCTGGCACTCAACATAGTAGGTGATGCAT |
| Tuber | SRR866242.sra.1195674 | CGAGGAATAAGTGAACAGTTGCTGGCACTCAACACAGTAGGTGATGCATC |
| Tuber | SRR866242.sra.13424756 | GGAATAAGTGAACAGTTGCTGGCACTCAACATAGTAGGTGATGCATCTGA |
| Tuber | SRR866242.sra.256842 | GAATAAGTGAACAGTTGCTGGCACTCAACATAGTAGGTGATGCATCTGAT |
| Tuber | SRR866242.sra.2181986 | GAATAAGTGAACAGTTGCTGGCACTCAACACAGTAGGTGATGCATCTGAT |
| Tuber | SRR866242.sra.3990289 | GTGAACAGTTGCTGGCACTCAACACAGTAGGTGATGCATCTGATTCTCCT |
| Tuber | SRR866243.sra.14812011 | TGAACAGTTGCTGGCACTCAACATAGTAGGTGATGCATCTGATTCTCCTA |
| Tuber | SRR866243.sra.9153005 | GTTGCTGGCACTCAACATAGTAGGTGATGCATCTGATTCTCCTACATCAG |
| Tuber | SRR866243.sra.2354693 | CTGGCACTCAACACAGTAGGTGATGCATCTGATTCTCCTACATCAGCTCC |
| Tuber | SRR866250.sra.6728833 | GCCAGCACACGAGGAATAAGTGAACAGTTGCTGGCACTCAACATAGTAGG |
| Tuber | SRR866250.sra.4991040 | CGAGGAATAAGTGAACAGTTGCTGGCACTCAACATAGTAGGTGATGCATC |
| Tuber | SRR866250.sra.5005162 | CGAGGAATAAGTGAACAGTTGCTGGCACTCAACATAGTAGGTGATGCATC |
| Tuber | SRR866250.sra.7677174 | CGAGGAATAAGTGAACAGTTGCTGGCACTCAACACAGTAGGTGATGCATC |
| Tuber | SRR866250.sra.10734484 | AGGAATAAGTGAACAGTTGCTGGCACTCAACATAGTAGGTGATGCATCTG |
| Tuber | SRR866250.sra.16815557 | GAATAAGTGAACAGTTGCTGGCACTCAACATAGTAGGTGATGCATCTGAT |
| Tuber | SRR866266.sra.3738582 | CAGCATACGAGGAATAAGTGAACAGTTGCTGGCACTCAACACAGTAGGTG |
| Tuber | SRR866266.sra.10394873 | CAGCACACGAGGAATAAGTGAACAGTTGCTGGCACTCAACATAGTAGGTG |
| Tuber | SRR866266.sra.15967051 | CACGAGGAATAAGTGAACAGTTGCTGGCACTCAACATAGTAGGTGATGCA |
| Tuber | SRR866266.sra.61808 | CGAGGAATAAGTGAACAGTTGCTGGCACTCAACATAGTAGGTGATGCATC |
| Tuber | SRR866266.sra.4230581 | TAAGTGAACAGTTGCTGGCACTCAACACAGTAGGTGATGCATCTGATTCT |

The 200 bp exonic sequence crossing the exon-exon boundary of the target intron: GTGACAACTAGCTTTCAAAGAGATCATTACGATCCTAGGCCATCTACATTCAGAGACAGGGCCAGCACACGAGGAATAAGTGAACAGTTGCTGGCACTCA<intron>ACATAGTAGGTGATGCATCTGATTCTCCTACATCAGCTCCACGAATACCATCACCTCCAATGAGTCCAGTGACAACTAGCTTTCAAAGAGATCATTACGA.

A read is regarded as being mapped crossing a boundary only when the number of mapped nucleotides on either side is ≧5.

Table S2. The WGS reads mapped crossing the boundaries of the two duplicates.





The 200 bp sequence crossing Site 1:

ataagagtatctgaagatgaaaatgtccttacaataggtaatatctaaagaataattcccgtattgtttattcacatataaattgagtatatatctggac<Site1>attagtttaggcttaatgaagaacttgttcaaattttttattggttgcgatctcttcttccttttttttgcatatttacaactctacatgtaaactatgt

The 200 bp sequence crossing Site 2:

GCTTTCAAAGAGATCATTACGATCCTAGGCCATCTACATTCAGAGACAGGGCCAGCACACGAGGAATAAGTGAACAGTTGCTGGCACTCAgtaagcttga<Site2>attagtttaggcttaatgaagaacttgttcaatttttttattggttgcgatctcttcttcctttttttgcatatttacaactctacatgtaaactatgtt

The 200 bp sequence crossing Site 3:

GCTTTCAAAGAGATCATTACGATCCTAGGCCATCTACATTCAGAGACAGGGCCAGCACACGAGGAATAAGTGAGCAGTTACTGGCACTCAGTAAGCTTGA<Site3>ATTCAGGAAATTCTTTTTGATTCTAAACTACATTGGGAGgtaaatttagcatttattcaaatatttactttgtgacttctatattgctacaagttggtat

Intronic sequences are presented in lowercase, and exonic sequences are in uppercase. A WGS read is regarded as being mapped crossing a target site only when the number of mapped nucleotides on either side is ≧5.

| Read IDs | Read Sequences |
| --- | --- |
| Site 1 |  |
| SRR307668.sra.20911835 | TATTGTTTATTCACATATAAATTGAGTATATATCTGGACATTAGTTTAGGCTTAATGAAGAACTTGT |
| SRR307703.sra.11867961 | TATTCACATATAAATTGAGTATATATCTGGACATTAGTTTAGGCTTAATGAAGAACTTGTTCA |
| SRR307703.sra.6842705 | ATTCACATATAAATTGAGTATATATCTGGACATTAGTTTAGGCTTAATGAAGAACTTGTTCAAATTT |
| SRR307686.sra.8916980 | ATTCACATATAAATTGAGTATATATCTGGACATTAGTTTAGGCTTAATGAAGAACTTG |
| SRR307686.sra.19641854 | TAAATTGAGTATATATCTGGACATTAGTTTAGGCTTAATGAAGAACTTGCTCAAATTTTTTA |
| SRR307691.sra.14196321 | CACATATAAATTGAGTATATATCTGGACATTAGTTTAGGCTTAATGAAGAA |
| SRR307691.sra.5757195 | AATTGAGTATATATCTGGACATTAGTTTAGGCTTAATGAAGAACTTGTTCAAATTTTTTAGC |
| SRR307691.sra.2382222 | CTTTGAGTATATATCTGGACATTAGTTTAGGCTTAATGAAGAACTTGTTCAAATTTTTTATT |
| SRR307691.sra.5228627 | ATATATCTGGACATTAGTTTAGGCTTAATGAAGAACTTGTTCAAATTTTTTATTGGTTGCCA |
| SRR307691.sra.19912973 | TATATCTGGACATTAGTTTAGGCTTAATGAAGAACTTGTTCAAATTTTTTATTGGTTGCGAC |
| SRR307611.sra.16527052 | TCTGGACATTAGTTTAGGCTTAATGAAGAACTTGTTCAAATTTTTTATTGGTTGCGATCT |
| SRR307666.sra.15423643 | ATTGAGTATATATCTGGACATTAGTTTAGGCTTAATGAAGAACTTGTTCAAATTTTTTAT |
| SRR307648.sra.15860875 | TATTCACATATAANTTGAGTATATATCTGGACATTAGTTTAGGCTTAATGAAGAACTTGTTCA |
| SRR307648.sra.14591446 | ATTCACATATAAATTGAGTATATATCTGGACATTAGTTTAGGCTTAATGAAGAACTTGTTCAA |
| SRR307701.sra.46025 | GAATAATTCCCGTATTGTTTATTCACATATAAATTGAGTATATATCTGGACATTAGTTTAGGCTTAA |
| SRR307701.sra.2514979 | ATATATCTGGACATTAGTTTAGGCTTAATGAAGAACTTGTTCAAATTTTTTATNGGTTGCGATC |
| SRR307701.sra.17955035 | TCTGGACATTAGTTTAGGCTTAATGAAGAACTTGTTCAAATTTTTTATTGGTTGCGATCTCTTC |
| Site 2 |  |
| SRR307668.sra.2540821 | TTCAGAGACAGGGCCAGCACACGAGGAATAAGTGAACAGTTGCTGGCACTCAGTAAGCTTGAATTAG |
| SRR307668.sra.10052925 | ACACGAGGAATAAGTGAACAGTTGCTGGCACTCAGTAAGCTTGAATTAGTTTAGGNTTAATGAAG |
| SRR307703.sra.18572963 | GCACTCAGTAAGCTTGAATTAGTTTAGGCTTAATGAAGAACTTGTTCAATTTTTTTATTGGTT |
| SRR307686.sra.6953648 | GCTTGAATTAGTTTAGGCTTAATGAAGAACTTGTTCAATTTTTTTATTGGTTGCGATCTCTT |
| SRR307691.sra.15230150 | ACAGGGCCAGCACACGAGGAATAAGTGAACAGTTGCTGGCACTCAGTAAGCTTGAATTAGAT |
| SRR307691.sra.10162142 | GAACAGTTGCTGGCACTCAGTAAGCTTGAATTAGTTTAGGCTTAATGAAGAACTTGTTCATT |
| SRR307691.sra.14158436 | CAGTTGCTGGCACTCAGTAAGCTTGAATTAGTTTAGGCTTAATGAAGAACTTGTTCAATTGC |
| SRR307611.sra.11941603 | GGGCCAGCACACGAGGAATAAGTGAACAGTTGCTGGCACTCAGTAAGCTTGAATTAGTTT |
| SRR307611.sra.1524825 | ACGAGGAATAAGNGAACAGTTGCTGGCACTCAGTAAGCTTGAATTAGTTTAGGCTTAATG |
| SRR307611.sra.11402320 | GAGGAATAAGTGAACAGTTGCTGGCACTCAGTAAGCTTGAATTAGTTTAGGCTTAATGAA |
| SRR307666.sra.4177666 | GGCCAGCACACGAGGAATAAGTGAACAGTTGCTGGCACTCAGTAAGCTTGAATTAGTTTA |
| SRR307666.sra.14915312 | GCCAGCACACGAGGAATAAGTGAACAGTTGCTCGCACTCAGTAAGCTTGAATTAGTTTAG |
| SRR307666.sra.4096851 | GCACTCAGTAAGCTTGAATTAGTTTAGGCTTAATGAAGAACTTGTTCAATTTTTTTATTG |
| SRR307648.sra.15049691 | TGAACAGTTGCTGGCACTCAGTAAGCTTGAATTAGTTTAGGCTTAATGAAGAACTTGTTCAAT |
| SRR307648.sra.2722934 | GAACAGTTGCTGGCACTCAGTAAGCTTGAATTAGTTTAGGCTTAATGAAGAACTTGTTCAATTTTT |
| SRR307648.sra.18667884 | AGTAAGCTTGAATTAGTTTAGGCTTAATGAAGAACTTGTTCAATTTTTTTATTGGTTGCGATCTCT |
| SRR307701.sra.6383738 | ACGAGGAATAAGTGAACAGTTGCTGGCACTCAGTAAGCTTGAATTAGTTTAGGCTTAATGAAGAACT |
| SRR307701.sra.18876344 | GAGGAATAAGTGAACAGTTGCTGGCACTCAGTAAGCTTGAATTAGTTTAGGCTTAATGAAGAAC |
| Site 3 |  |
| SRR307611.sra.17511416 | ACACGAGGAATAAGTGAGCAGTTACTGGCACTCAGTAAGCTTGAATTCAGGAAAGCCTTTT |
| SRR307611.sra.1836556 | AGTTACTGGCACTCAGTAAGCTTGTATTCAGGAAATTCTTTTTGATTCTAAACTACATTGGGAGGTAAA |
| SRR307611.sra.337437 | CACTCAGTAAGCTTGAATTCAGGAAATTCTTTTTGATTCTAAACTACATTGGGAGGTAAAT |
| SRR307611.sra.7788356 | CAGTAAGCTTGAATTCAGNAAATTCTTTTTGATTCTAAACTACATTGGGAGGTAAATTTAGCATTTATT |
| SRR307611.sra.12059209 | AGTAAGCTTGAATTCAGGAAATTCTTTTTGATTCTAAACTACATTGGGAGGTAAATTTAGC |
| SRR307648.sra.19729397 | CCAGCACACGAGGAATAAGTGAGCAGTTACTGGCACTCAGTAAGCTTGAATTCAGGAAATTCTTTT |
| SRR307648.sra.2812439 | GCTTGAATTCAGGAAATTCTTTTTGATTCTAAACTACATTGGGAGGTAAATTTAGCATTTATT |
| SRR307666.sra.7935592 | GGGGCCAGCACACGAGGAATAAGTGAGCAGTTACTGGCACTCAGTAAGCTTGAATTCAGGAAA |
| SRR307666.sra.2487878 | CAGCACACGAGGAATAAGTGAGCAGTTACTGGCACTCAGTAAGCTTGAATTCAGGAAATTCTT |
| SRR307666.sra.9541505 | CAGTAAGCTTGAATTCAGGAAATTCTTTTTGATTCTAAACTACATTGGGAGGTAAATTTAGCATTT |
| SRR307668.sra.15396543 | AGAGACAGGGCCAGCACACGAGGAATAAGTGAGCAGTTACTGGCACTCAGTAAGCTTGAATTCAGGA |
| SRR307668.sra.18852112 | AAGTGAGCAGTTACTGGCACTCAGTAAGCTTGAATTCAGGAAATTCTTTTTGATTCTAAACTACA |
| SRR307668.sra.14684307 | AGTTACTGGCACTCAGTAAGCTTGAATTCAGGAAATTCTTTTTGATTCTAAACTACATTGGGAGGTA |
| SRR307668.sra.874384 | TGGCACTCAGTAAGCTTGAATTCAGGAAATTCTTTTTGATTCTAAACTACATTGNGAGGTAAATTTA |
| SRR307686.sra.18388025 | CAGTTACTGGCACTCAGTAAGCTTGAATTCAGGAAATTCTTTTTGATTCTAAACTACA |
| SRR307691.sra.2581471 | TAAGTGAGCAGTTACTGGCACTCAGTAAGCTTGAATTCAGGAAATTCTTTTTGATTCTAAAA |
| SRR307703.sra.17318168 | CACACGAGGAATAAGCGAGCAGTTACTGGCACTCAGTAAGCTTGAATTCAGGAATNTCTTTTT |
| SRR307703.sra.13140955 | TAAGTGAGCAGTTACTGGCACTCAGTAAGCTTGAATTCAGGAAATTCTTTTTGATTCTAAACTACAT |
